# News and Notes

**Published:** 1908-08

**Authors:** 


### NEWS AND NOTES

Dr. J. E. Flowers has left for Denver, Col.

---

Dr. and Mrs. W. S. Elkin spent some time at Tate Springs, and paid a short visit to New York during the past month.

---

Dr. John F. Denton has just returned from a professional visit to Dalton, Ga.

---

Dr. and Mrs. R. T. Dorsey have returned from a visit to Fayetteville.

---

Dr and Mrs. Giddings have left for a short visit to his old home in Virginia before sailing for Europe.

---

Dr. Stephen T. Barnett has returned from a recent trip to Clinton, S. C.

---

Dr. and Mrs. Michael Hoke are spending several days at Wrightsville Beach.

---

Dr. and Mrs. Wesley Taylor left recently for a visit to Chicago and other Western cities.

---

Dr. and Mrs. Arch Avary have returned from an enjoyable stay at Wrightsville Beach.

---

The many friends of Dr. Toepel will be glad to learn that he is at last able to leave his sick room and is rapidly convalescing from his recent severe spell of typhoid.

---

Many members of the profession took advantage of the opportunity afforded by the mid-summer meeting of the First Congressional District Medical Society at Savannah, to visit Tybee's beach to enjoy the traditional "Savannah hospitality."

---

Dr. and Mrs. Bates Block have returned from an extended tour of Europe, where they were most delightfully entertained by friends in both France and Germany.

During the third week in June the death rate in London was the lowest on record. It was only 10.8 per 1,000.

---

Dr. Floyd W. McRae has resigned from the staff of the Grady Hospital and Dr. W. B. Armstrong has been elected to fill his position as surgeon.

---

The Ocmulgee Medical Association convened at Cochran. Ga,, July 21. After the scientific meeting, a very enjoyable banquet was held.

---

The new Baptist Tabernacle Infirmary has been opened and now has sixty-five rooms, including three wards of ten beds each.

---

The entire staff of resident physicians of the Grady Hospital has resigned on account of the stringent rules. The resignations were accepted and other internes were selected to fill the vacancies.

---

The number of cases of appendicitis treated in the public hospitals of Prussia is said to have increased from 8,412 in 1903 to 16,781 in 1906.

---

Dr. S. A. Korpf, of New York City, has been elected Professor of Phthsiosio-Therapy to fill the newly created chair at the New York Post Graduate Medical School and Hospital.

---

The proposition of the Atlanta Medical Colleges to be admitted as part of the University of Georgia, was decided by the meeting of the board of trustees for the present, but a committee was appointed to make an investigation as to the advisability of making such a change.

---

Dr. Willis Westmoreland, Dr. R. R. Kime, Dr. F. G. Hodgson, Dr. Claud Smith, Dr. Barnett, Dr. Paulin and others appeared before the Legislature and made interesting speeches in support of the bill for a state sanatarium for incipient tuberculosis.

Dr. and Mrs. E. D. Richardson are spending some time at Borden-Wheeler Springs.

The Medical profession of Atlanta and vicinity learns with much pleasure, that Mr. E. Anthony, formerly of Brannen & Anthony, has purchased the Whitaker-Coursey Drug Company's business, and in the future will conduct same at 29 Marietta street, under the name of Anthonys' Pharmacy.

---

The Old Dominion Journal of Medicine and Surgery announces a change of management, beginning with the July issue. The new managers have rejuvinated this journal and have a long list of distinguished collaborators, and we wish for them success in their undertaking. We note with pride the increased interest now being taken in Southern medical journals and *pari passu* with this interest an improvement in the quality of journals.

---

The following were elected by the state society as the board of medical examiners of the State of North Carolina for six years: Drs. James L. Nicholson, Richlands; Henry H. Dodson, Greensboro; Lewis B. McBrayer, Asheville; William W. Mackenzie, Salisbury; Benjamin K. Hays, Oxford; John C. Rodman, Washington, and John Bynum, Winston-Salem.

---

### STATE SANATARIUM FOR TUBERCULOSIS.

We note with great pleasure the passage of the bill providing for a sanatarium for the treatment of tubercular patients. This bill was introduced last year by Drs. Whitely and Frye and has now passed the lower house by a vote of 140 to 14. This large majority seems particularly incouraging as the bill provides for the appropriation of $25,000 for the sanatarium and it is sincerely hoped that the Senate will concur with the House as Georgia is in much need of such an institution.

---

### GIBBS' PRIZE ESSAY.

The New York Academy of Medicine announced that the sum of one thousand dollars will be awarded to the author of the best essay in competition for the above mentioned prize.

The subject of the essay, as stated, shall be, "The Etiology, Pathology and Treatment of the Diseases of the Kidney."

Essays must be presented on or before October 1st, 1909.

The three subjects mentioned in the title as above given, need not be treated with uniform fullness, but new discovery or fruitful research will be considered the standard of merit.

Each essay must be in English, typewritten, designated by a motto, or device, and accompanied by a sealed envelope, bearing the same motto, or device, which shall contain the name and address of the author.

No envelope will be opened except that which accompanied the successful essay.

The Academy reserves the right, according to the direction of the donors, not to award the prize if no essay shall be deemed worthy of it.

The Academy will return the unsuccessful essays, if claimed by their respective authors, or by authorized agents, within six months.

An essay must show originality in order to obtain the prize.

The competition is open to the members of the regular medical profession of the United States.

The original of the successful essay shall be the property of the Academy, and, according to the deed of gift, will be published in its Transactions.

The essays shall be transmitted to the committee of the New York Academy of Medicine on the Edward N Gibbs memorial prize.

John A. Wyeth, President.

### A FORE-CAST.

Among the original articles to appear in early numbers of the Journal-Record of Medicine are the following: "Some Aspects of Intestinal Autointoxication, with Report of Two Cases Showing Unusual Skin Manifestations." By Dr. Lewis M. Gaines, Atlanta, Ga. "Hydatidiform Mole, (Myoma Chorii), with Report of a Case." By Dr. J. B. Cranmer, Wilmington, N. C. "Reciprocity Between States." By Dr. A. A. Kent, Lenior, N. C. "Importance of a Thorough Knowledfe of Bilology, Bacteriology and the Circulation of the Blood for the Successful Application of Serum Therapy." By Dr. J. C. Grady, Kenly, N. C. "Report of a Case of Suppurative Leiomyoma of the Stomach." By

Dr. Whatley W. Battey, Jr., August Ga. "Abscess of the Brain." By Dr. R. G. Buckner, Aheville, N. C. "Empyema—Etiology, Symptoms, Treatment and When to Perform Thoracotomy, with Report of Cases." By Dr. John T. Burrus, High Point, N. C. "A Report of Cases Treated with Ichthyolated Emulsion Compound." By Dr. John Roy Williams, Greensboro, N. C. "Quacks." By M. A. Clark, M. D., Macon, Ga. "Chronic Stomach Ulcers; Its Surgical Treatment." By Edward G. Jones, A. B., M. D., Professor of Surgery in the Atlanta School of Medicine. "The Treatment of the Morphine Habit in General Practice." By Geo. H. Lehman.

---

### REGULAR MEETING FULTON COUNTY MEDICAL SOCIETY, MAY 21, 1908.

REPORTED BY DR. R. R. DALY.

Dr. E. C. Davis made some "Remarks upon Obstetrical Nursing," largely in protest against incompetent, dirty negro nurses who assume charge of patients in the puerperal state.

Dr. Archibald Smith spoke of some obstetricians who boasted of never making post-partum visits and of never using a basin of antiseptic solution. The results were deplorable, but because they were common, they were allowed to continue. He advocated a hospital, if possible, and always some trained, clean nurse to care for the mother.

Dr. Willis Jones remarked that the increased morbidity and general difficulty in cities was largely due to greater amount of gonorrhoea. He said there was an expression in France, "One child, sterility," due to the great amount of infection in that country. He advocated education of males as to the danger of old and uncured gonorrhea before marriage.

Dr. H. R. Donaldson strongly reprobated negro nurses. He related a case of lateration which he had properly dressed and left. The "Mammy" came later, removed the dressing and covered the parts with salve of her own making.

Dr. Davis, in closing, agreed with Dr. Jones as to gonorrhoeal infection and said that 35 per cent. of post mortem morbidity was due to this cause and perhaps an equal per cent. of abortions.

He said that when there was no crisis, anybody could stay with the mother and care for her, but when the dangerous moments arrived—and no one could tell the time they would come—the negro was the very worst companion for the mother to have.

Dr. Willis Jones gave a "Review of Recent Literature of Appendicitis," which we published in full in the last issue of the Journal-Record of Medicine.

Dr. Claude Smith discussed the question of milk supply of Atlanta, and the methods in use for preventing the sale of impure milk. It is thought that tuberculosis among cows is not so frequent here as in other sections of the country. Dr. Smith urged the importance of fresh milk as the danger is greatly decreased when kept until stale. The danger of tuberculosis in milk is of small matter compared to the danger from other organisms which multiply in milk when allowed to remain warm.

Dr. Stirling asked Dr. Smith to state what per cent. of cows in this section had tuberculosis.

Dr. W. B. Armstrong said that the milk of Atlanta did not contain a large number of germs, but that a great menace was the careless manner in which milk is kept in the home and that the refrigerators usually do not maintain a sufficiently low temperature.

Dr. Hodgson said that the doctors of Atlanta were under many obligations to Dr. Smith for the excellent work he has done in improving the quality of milk.

Dr. Smith, replying to Dr. Stirling, said the percentage of tuberculosis was not accurately determined, but that the inspector from Washington has looked over a large heard of cows, and only found one cow which appeared to be tuberculius.

Dr. E. C. Cartledge read a paper on the "Treatment of Constipation."

Dr. E. G. Jones read a paper on "Gastric Ulcer." Discussed by Dr. Willis Jones and Dr. J. C. Olmstead.

Dr. W. S. Goldsmith reported the history of a patient who could not control the passage of gas from his bowels and had great difficulty in defecation.

Dr. Goldsmith also demonstrated rare specimens of dermoidcyst of the kidney. Undoubted evidence of a tooth and hair were found in the cyst.

Dr. Paullin had examined the kidney shown by Dr. Goldsmith, and thought there was a connection between the cyst and the pelvis of the kidney. There was only slight evidence of nephritis.

Dr. Willis Jones related history of patient pertoneal tuberculosis who appeared to be in a hopeless condition when operated upon, but most astonishing improvement had followed loparotomy.

